# *Chlamydia trachomatis* Genital Infections

**DOI:** 10.15698/mic2016.09.525

**Published:** 2016-09-05

**Authors:** Catherine M. O’Connell, Morgan E. Ferone

**Affiliations:** 1 Department of Pediatrics, University of North Carolina at Chapel Hill, Chapel Hill, North Carolina, USA.

**Keywords:** Chlamydia, urogenital, infection, epidemiology, reproductive morbidity, treatment

## Abstract

Etiology, transmission and protection: *Chlamydia trachomatis* is the leading cause of bacterial sexually transmitted infection (STI) globally. However, *C. trachomatis* also causes trachoma in endemic areas, mostly Africa and the Middle East, and is a leading cause of preventable blindness worldwide. Epidemiology, incidence and prevalence: The World Health Organization estimates 131 million new cases of *C. trachomatis* genital infection occur annually. Globally, infection is most prevalent in young women and men (14-25 years), likely driven by asymptomatic infection, inadequate partner treatment and delayed development of protective immunity. Pathology/Symptomatology: *C. trachomatis* infects susceptible squamocolumnar or transitional epithelial cells, leading to cervicitis in women and urethritis in men. Symptoms are often mild or absent but ascending infection in some women may lead to Pelvic Inflammatory Disease (PID), resulting in reproductive sequelae such as ectopic pregnancy, infertility and chronic pelvic pain. Complications of infection in men include epididymitis and reactive arthritis. Molecular mechanisms of infection: Chlamydiae manipulate an array of host processes to support their obligate intracellular developmental cycle. This leads to activation of signaling pathways resulting in disproportionate influx of innate cells and the release of tissue damaging proteins and pro-inflammatory cytokines. Treatment and curability: Uncomplicated urogenital infection is treated with azithromycin (1 g, single dose) or doxycycline (100 mg twice daily x 7 days). However, antimicrobial treatment does not ameliorate established disease. Drug resistance is rare but treatment failures have been described. Development of an effective vaccine that protects against upper tract disease or that limits transmission remains an important goal.

## INTRODUCTION

*Chlamydia trachomatis* infections are the most commonly reported sexually transmitted bacterial infections in the US and globally. Ascending infection may result in infertility, ectopic pregnancy and chronic pelvic pain in some women. Despite widespread screening and treatment programs, the *Chlamydia* epidemic continues unabated with yearly increases in the number of reported cases. *C. trachomatis* is a gram-negative obligate intracellular pathogen with a unique developmental cycle that infects ocular, genital and respiratory tissues. Intriguingly, chlamydial serovars display specific tropisms for different mucosal sites but the molecular mechanisms controlling these processes are not fully understood. *C. trachomatis* can be classified into 15 serovars (genovars) 1 based on antigenic variation in the major outer membrane protein (MOMP) encoded by *ompA* 2. Serovars A-C are associated with trachoma, serovars D-K are most commonly with urogenital infection and serovars L1-L3 represent strains causing invasive lymphoma granuloma venereum (LGV). Although we have developed insights into how this bacterium infects and establishes a protected niche within epithelial cells using cell culture models, and have established animal models of *in vivo* infection, we lack information regarding the mechanisms that promote ascension and elicit damaging immunopathology in humans. Consequently, we are challenged to define chlamydial markers of virulence or biomarkers of host disease that could predict risk for severe reproductive sequelae and improve targeted screening and treatment. Chlamydial research is entering a period of rapid expansion with the advent of molecular epidemiology techniques, abundant genome sequences, and new approaches for effective genetic manipulation. With new tools to investigate the pathogenic mechanisms driving chlamydial disease in humans we hope to see accelerated progress towards an effective vaccine. In this review we provide an overview of current knowledge regarding epidemiology, disease outcomes and effective treatment of chlamydial genital tract infection. We also explore potential mechanisms facilitating *C. trachomatis* infection of genital mucosa identified via bioinformatics and other molecular approaches.

## EPIDEMIOLOGY

*C. trachomatis* is the leading cause of bacterial sexually transmitted infection (STI) in the world. However, in endemic areas, mostly in Africa and the Middle East, *C. trachomatis* also causes trachoma, a leading cause of preventable blindness worldwide. The World Health Organization estimated a global prevalence of chlamydia at 4.2% (95% uncertainty interval: 3.7-4.7) among women aged 15-49 years for 2012 3. These figures correspond to an estimated 131 million new cases of chlamydia (100-166 million) 3. The majority of infections are observed within the Western Pacific Region and the Region of the Americas. Within the USA, 1,441,789 chlamydial infections were reported to CDC in 2014 4. Most infected men and women are either asymptomatic or minimally symptomatic and diagnosis occurs after screening or because a contact is symptomatic.

Rates of reported cases of chlamydia are highest among adolescents and young adults aged 15-24 years. In 2014, the rate among 15-19 year olds was 1,804.0 cases per 100,000 and the rate among 20-24 year olds was 2,484.6 cases per 100,000 4. Prevalence is relatively high when compared with other bacterial STIs because asymptomatically infected individuals may not seek treatment and repeat infection after single dose therapy is common. Infection is more frequently reported in young women rather than young men 4,5. Additional predictors of incident chlamydial infection in young women include single marital status, having a new sex partner or concurrent partnerships, smoking and associated signifiers of socioeconomic status, having gonorrhea or bacterial vaginosis, and presence of carcinogenic human papillomavirus 5,6,7,8,9.

## GENITOURINARY TRACT INFECITON, DISEASE and REPRODUCTIVE SEQUELAE

Symptoms of genital *C. trachomatis* infection in women, when present, include changes in vaginal discharge, intermittent, intermenstrual and/or post-coital bleeding. *C. trachomatis* can also infect the urethra and some patients may present with symptoms of urinary tract infection (frequency and dysuria) 10. Mucopurulent endocervical discharge, easily induced endocervical bleeding, or edematous ectopy are clinical signs that may be observed upon exam 11. Untreated, infection may persist for up to 4 years 12 although spontaneous clearance of infection after diagnosis has been described 13, suggesting development of some degree of protective immunity.

Infection may ascend from the cervix, resulting in endometritis and salpingitis. Chlamydial PID can present as pelvic or lower abdominal pain with cervical motion tenderness or uterine or adnexal tenderness at exam 14 but even upper genital tract infection may be asymptomatic 15. In a high-risk population, 2% to 5% of untreated women developed PID within a ~2-week elapse between testing positive for *C. trachomatis* and returning for treatment 16,17. Repeated chlamydial infection has been associated with PID and other reproductive sequelae 16,18. A direct assessment of the risk for infertility after untreated *C. trachomatis* infection has not been performed but it has been determined that up to 18% of women may develop infertility after symptomatic PID of any cause 19.

*C. trachomatis* genital tract infection can also negatively impact pregnancy. Prior chlamydial infection is associated with elevated risk for ectopic pregnancy 20,21. *C. trachomatis* infection has been associated with spontaneous abortion, stillbirth and preterm delivery 22,23,24. *C. trachomatis* can also be transmitted to a neonate during delivery via contact with infected cervix tissue and secretions leading to infection of mucous membranes of the eye, oropharynx, urogenital tract, and rectum. Infection may be asymptomatic in these locations. *C. trachomatis* conjunctivitis that develops 5-12 days after birth is the most common presentation 25 but *C. trachomatis* also can cause a subacute, afebrile pneumonia with onset at ages 1-3 months 26. These outcomes are best avoided by screening and treatment prior to delivery.

In addition to serious reproductive consequences such as infertility, ectopic pregnancy and chronic pelvic pain *C. trachomatis* has also been proposed as a possible risk factor for cervical cancer. Human Papillomavirus (HPV) is a known cause of cervical cancer, but exposure to HPV does not necessarily result in the development of HPV-related cervical cancer. Persistent, high-risk HPV infections are more likely to progress to squamous cell carcinoma (SCC) or invasive cervical cancer (ICC) in the presence of identified cofactors including smoking, behavioral factors, age, genetic background and individual immune variation 27. Chronic cervical infection by *C. trachomatis* has been proposed as a cofactor based on detection of chlamydial DNA in HPV-associated lesions 28 and studies correlating the presence of anti-CT antibodies with risk for ICC or SCC 29. A recent meta analysis of 22 studies (19 retrospective, 3 prospective) determined that *C. trachomatis* was significantly linked to increased cervical cancer risk prospectively (OR = 2.21, 95% CI: 1.88-2.61, P < 0.001), and retrospectively (OR = 2.19, 95% CI: 1.74-2.74, P < 0.001) 30. The overlap in factors that contribute to HPV and chlamydial infection such as age 31 and number of sex partners 32 makes it challenging to determine if this association reflects concurrent infection or if chlamydial infection acts indirectly to facilitate HPV infection and/or promote HPV persistence. However, *C. trachomatis* infection was identified as an independent predictor of cervical cancer in 11 of these studies (OR = 1.76, 95% CI: 1.03-3.01, P = 0.04) with a multivariate logistic regression analysis adjusted for HPV and age 30.

Recent studies indicate that the origin of high-grade serous carcinomas is the fallopian tube 33. Precursor lesions that contain evidence of DNA damage and p53 mutations 34 have been detected in the fimbriated portion of the tubes of women with BRCA mutations 35. This association has prompted some gynecologic oncologists to advocate prophylactic bilateral salpingectomy for low risk women, coincident with benign gynaecologic surgery, e.g. hysterectomy or tubal ligation, as a primary preventive for ovarian cancer 36. Primary fallopian tube carcinomas have described in patients with chronic PID 37 and infertility is a known risk factor for epithelial ovarian cancer (reviewed in 38). A pilot study initially supported an association with anti-chlamydial antibody and ovarian cancer 39 but a subsequent study in a larger cohort could not confirm this finding 40. Anti-chlamydial IgG has been associated with type II ovarian cancer (P=0.002) in women with plasma samples obtained >1 year prior to diagnosis (n=7) 41. Positive 42 and negative 43 reports of the detection of chlamydial DNA in tumor tissue specimens have been published. Should future studies validate these still equivocal findings, women with a history of ascended chlamydial infection may be at increased risk for neoplasia.

*C. trachomatis* is the most common cause of nongonococcal urethritis in men. As in women, infections are often asymptomatic (40% to 96%) 44,45. The incubation period is variable but is typically 5 to 10 days after exposure. When men have symptoms, they may present with a mucoid or watery urethral discharge, and complain of dysuria. Their discharge may be scanty, clear or only observed after milking the urethra 46. Chlamydial infection may result in inflammation of the epididymides and testes. *C. trachomatis* is one of the most frequent pathogens in epididymitis among sexually active men <35 years of age. Symptoms of acute epididymitis include testicular pain and tenderness, hydrocele and epididymal swelling 47. Approximately 1% of men with nongonococcal urethritis develop reactive arthritis, and about one-third of these patients display the complete reactive arthritis triad previously termed Reiter syndrome (arthritis, uveitis, and urethritis) 48,49. Chlamydial nucleic acids have been detected in synovial tissues from patients with sexually transmitted reactive arthritis 50,51. The potential for *C. trachomatis* to cause chronic prostatitis and it’s potential to negatively impact male infertility is controversial (reviewed by 52).

Chlamydial proctitis, inflammation of the distal rectal mucosa, occurs primarily in men who have sex with men (MSM) who engage in receptive anal intercourse. In this group, infection is not uncommon and can be caused by D-K and L serovars. The presentation and severity of disease depends on the infecting chlamydial serovars. L1, L2 and L3 serovars of *C. trachomatis* cause LGV. In tropical and sub tropical regions, LGV infections are associated with urogenital ulceration and invasion of the lymphatic system in both men and women, which can result in bubo formation, fistulae, fibrosis and rectal stenosis. Outbreaks of anorectal disease caused by the L1-3 serovars have been reported amongst European and North American MSM, particularly those who are HIV-infected 53. In contrast to infection with serovars associated with genital tract infection, these infections are frequently symptomatic. Symptoms include anorectal pain, discharge, tenesmus, rectal bleeding and constipation, and are often accompanied by fever 54. Lack of treatment may result in strictures and severe scarring.

## *CHLAMYDIA TRACHOMATIS* - GENITAL TRACT PATHOGEN

*C. trachomatis* is a strict intracellular pathogen with a unique biphasic lifecycle. Upon attachment, infectious elementary bodies (EB) stimulate uptake into epithelial cells where they differentiate into vegetative reticulate bodies (RB) to grow and divide within a membrane bound, host-derived parasitophorous vacuole called an inclusion. Within 8-12 divisions 55, differentiation to EB is initiated and the cycle is complete when the cell releases the contents of the inclusion to attach to adjacent cells and reinitiate the cycle 56. Chlamydial appropriation and exploitation of host cell machinery during invasion, inclusion formation and development is fundamental to replicative success. However, this "hijack" of cellular processes and intermediates triggers pro-inflammatory signaling pathways that drive innate cell influx with cytokine and chemokine release. For a subset of women with ascending infection the ultimate outcome is severe immunopathology and fibrosis leading to tubal occlusion (reviewed by Haftner 57). Similarly, processes that support chlamydial multiplication intracellularly can predispose the host cell towards transformation. Much of our current understanding of the roles that specific chlamydial effectors and their interactive host partners play in these processes is derived from cell systems 58,59,60,61 and animal models 62,63. Candidate receptors that promote chlamydial attachment to susceptible cells have been identified 64,65,66. Not unsurprisingly for a microorganism that interacts with its host cell across the plasma membrane at attachment and entry and with the inclusion membrane during the remainder of the developmental cycle, secretion systems represent a significant portion of the chlamydial genome. Type III secretion is important to effective cellular entry with a key role for the secreted effector TARP as well as potential accessory proteins (reviewed in 67). Furthermore, chlamydiae secrete a strikingly large number of proteins (Inc) to the inclusion membrane that play critical roles in membrane fusion, promote nutrient acquisition, avoidance of autophagy, engagement of innate signaling etc. 68. Global mapping of the Inc-Human interactome via affinity purification-mass spectroscopy (AP-MS) has identified associations or engagement of host proteins during all aspects of the chlamydial developmental cycle and revealed a previously unappreciated role for IncE in disruption of retromer trafficking via sequestration of SNX5/6 69. A recent review of the role(s) of these proteins and their cellular targets has been published which discusses these interactions in great detail, revealing the extent to which chlamydiae re-engineer the cell to support their multiplication 61.

Defining a spectrum of virulence for *C. trachomatis* strains in the context of genital tract infection has been challenging. Identifying clinical features that predict ascending infection and disease development in susceptible individuals is problematic because even PID may be asymptomatic 15. Disease outcome is also influenced by the genetic predisposition of an infected individual (~40%) 70. Genome studies and innovative efforts to better characterize infection kinetics and host response may prove useful, while genetic manipulation of strains will provide a direct route to examining the contribution of candidate virulence loci to infectivity, transmission, immunopathology or cancer.

The sequence of *C. trachomatis* D/UW-3/Cx was published in 1988 71 and since then many more strains have been sequenced (137 complete or partial genomes in Genbank, July 2016). Overall, *C. trachomatis* strains are strikingly similar with respect to size, GC content, similarity and synteny with near overlap between their core and pan genomes 72,73,74. This is preserved at the level of the resident plasmid with concordance of chromosome and plasmid phylogenies 75. Plasmid host range is highly restricted because shuttle vectors constructed from plasmids obtained from *C. muridarum*, *C. trachomatis* LGV or trachoma plasmids could not be stably transformed outside their lineage 76. The *C. trachomatis* phylogenetic tree parallels tissue-tropic groupings, LGV strains splitting first from urogenital and trachoma strains that subsequently diverged 75. Strains causing genital infections form two clades 72,73, one encompassing the most commonly isolated serovars E and F 77, with a second group comprised of serovars D, G-K. Within the clades, genetic exchange or recombination within the *ompA* locus has been detected where the major proportion of the genome remains consistent with the clade even when *ompA* type is discordant 72,78. Sequencing of trachoma strains endemic in Australian aboriginal communities determined that these isolates are more closely related to urogenital strains than to the classic trachoma lineage with the exception of their *ompA* and *pmpEFGH* loci 79 indicating that trachoma lineages have arisen from urogenital strains more than once.

Interestingly, these trachoma strains retain a functional TrpA 79. Previously, truncations of *trpA* were considered an important feature of ocular strains, contrasting with urogenital strains that express functional TrpA 80 and synthesize tryptophan if provided with indole 81. The potential that urogenital strains might be able to avoid IFN-γ -mediated chlamydial killing, which acts via IDO-induced tryptophan degradation 82, via cross-feeding from indole-producing commensals of the genital tract was thought to reflect niche expansion. The observation that trachoma strains may have arisen more than once, suggests that mutation of *trpA* could be pathoadaptive with respect to overall metabolism 83. More recently, the potential that TARP, a type III effector critically important in chlamydial entry and the highly polymorphic surface proteins (Pmps) play important roles in tissue tropism has been described 64,79,84. Cell culture-based studies of invasive, lypmphotropic LGV strains suggested that they were capable of surviving within macrophages 85,86, and were less susceptible to IFN-γ 87. However, L2 strains are no better at resisting perforin-2 mediated killing by activated human macrophages than the urogenital serovars B or D 88. Bioinformatic approaches have been employed to identify highly polymorphic loci (*pmp*, *inc*, TARP), recombination hotspots, and loci under positive or purifying selection with the goal of identifying individual genes that contribute to tissue tropism and virulence in LGV and urogenital strains 74,78,89,90,91. Although this approach is unbiased and the sequences analyzed represent the range of natural variation compatible with successful occupation of this ecologic niche, teasing out the individual contributions of candidate virulence factors still requires functional and mechanistic studies.

Another approach to investigate virulence differences between urogenital strains is to identify *in vivo* phenotypes predicting superior pathogenic potential. The conserved plasmid that is present in nearly all strains of *C. trachomatis* and in *C. muridarum* plays an important, highly pleiotropic role in virulence. Plasmid-deficient *C. muridarum* are attenuated in the murine model of genital tract infection because their ability to elicit damaging upper tract inflammation is reduced 92. Plasmid-deficient *C. muridarum* compete poorly with their plasmid-containing parent *in vivo* 93 and are less successful establishing oviduct infection 92,93. A plasmid-cured derivative of trachoma-causing *C. trachomatis* is attenuated in a non-human primate model of ocular infection 94. Cynomolgus macaques inoculated with strain A/2497P- displayed reduced inflammation and infection was cleared rapidly. In contrast, infection parameters did not differ significantly between *C. trachomatis* CTD153, a plasmid-cured derivative of the urogenital strain, D/UW-3/Cx and its parent when inoculated intravaginally in rhesus macaques 95. Similarly, accelerated clearance of infection by plasmid-deficient chlamydia is not observed in mice 92. It is possible that this phenotype reflects a plasmid-associated difference that contributes to tissue tropism. The chlamydial plasmid is also required for accumulation of glycogen within inclusions 96,97,98. Pgp4 is the plasmid-borne transcriptional regulator of the adjacent *pgp3* 99 and a conserved group of chromosomal loci, including *glgA*, which are differentially expressed in plasmid-deficient strains 98,100. *C. muridarum* *pgp3* mutants are attenuated *in vivo* 101, indicating that this protein likely plays an important role in chlamydial virulence. The mechanism(s) by which Pgp3 contributes to chlamydial pathogenesis remains unclear, although roles in TLR2 activation 102 and immune avoidance via binding of the antimicrobial peptide LL-37 have been proposed 103. These studies demonstrate that chlamydial virulence is not intrinsically linked to fitness and that chlamydiae coordinate expression of genes or pathways important for pathogenesis. Conditions that stress chlamydiae 104,105,106 result in distinctive and profound transcriptional changes, superimposed on the complex transcriptional program that regulates the chlamydial developmental cycle 107. TLR2 activation and PRCL transcription by *C. trachomatis* is reduced in cell culture when glucose is limited 100, indicating that regulatory networks could potentially modulate virulence effector expression during infection in response to environmental stressors.

Association of signs, symptoms, and serovar with chlamydial load in diagnostic samples has been investigated as a way to assess infection severity (reviewed by 108). Increased cervical burden correlated with ascending infection using endometrial biopsies to monitor upper tract infection 109. However, a recent attempt to associate the presence or absence of the plasmid with reproductive morbidities in women presenting with gynecologic complications or subfertility was unsuccessful because the prevalence of plasmid-deficient strains in the study population was too low 110. The feasibility for extensive evaluation of cervical infection, coordinating immunofluorescent, ultrastructural genomic or flow cytometric analysis of infected cervical cells recovered via cytobrush, has been demonstrated 111,112. Transcriptional profiling using blood obtained from women with PID or asymptomatic cervical infection suggests that a blood borne inflammatory signature could enable the identification of specific biomarkers of damaging host responses 113,114. Correlation of infecting strain with engagement of such biomarkers may also be a way to identify virulent strains. However, the requirement for large numbers of infected patients combined with the expense associated with molecular and/or immunologic assays may render studies of sufficient statistical power cost prohibitive.

Determining the mechanisms by which chlamydial infection contributes to cellular transformation is key to understanding its potential role in reproductive cancers. HPV can induce genetic instability via dysregulation of centrosome duplication and p53 suppression 115. Multiple phenotypes related to genetic instability have been observed in chlamydial infection, such as supernumerary centrosomes, abnormal spindle poles, multinucleation, and chromosomal segregation defects 116,117,118. Dysregulation of host centrosome duplication during chlamydial infection occurs at procentriole formation, requires host kinases Cdk2 and Plk4 and progression through S-phase 117. Chlamydial disruption of this host pathway does not impact generation of infectious progeny 117. However, chlamydiae also usurp host microtubule networks as they establish their intracellular niche. Initial trafficking to the centrosome along microtubules involves the recruitment of Src kinases to the inclusion membrane, where their interaction with inclusion membrane (Inc) proteins facilitates access to the microtubule network at the centrosome 116,119,120. Chlamydia then orchestrate reorganization of the host microtubule network via Inc protein IPAM (inclusion protein acting on microtubules) and host-encoded CEP170 into a scaffold to support and maintain the inclusion within the cell 121, at the apparent cost of further centrosomal abnormality. There is no direct evidence to indicate that such abnormalities directly mediate tumor initiation but centrosomal abnormalities are observed in early, pre-cancerous lesions, hinting of a contribution to tumor progression 122. Regardless, cytokinesis failure and/or centrosome overduplication normally activates the tumor supressor p53 pathway 123.

Chlamydial infection also inhibits cellular DNA damage repair pathways directly, leading to heritable defects 124. Chlamydial infection triggers formation of reactive oxidative species, which promotes double stranded DNA breaks (DSBs). Downstream DNA repair responses and DSB relevant cell-cycle checkpoints are overridden 124 because intracellular chlamydiae activate a host pathway that culminates in proteasomal degradation of p53 125,126. These events grant chlamydiae access to vital energy intermediates because p53 down-regulates the pentose phosphate pathway within it damage surveillance program 126. Infected cells continue to proliferate despite the damage they sustain 124. Thus, in the context of acute infection, chlamydiae successfully meet their metabolic requirements and preserve their cellular niche. Current understanding of the developmental cycle suggests that the damaged cell will be destroyed rather than transformed after inclusion lysis and bacterial release. However, infected cells are able to divide and pass genetic defects onto daughter cells in culture 116,127 and in mice 127. Furthermore, 3T3 cells infected and cured of chlamydia exhibit anchorage-independent growth and increased rates of colony formation compared to mock-infected 3T3s 127, suggesting that mutagenized cells could escape infection and initiate neoplasm. Cervical dysplasia has been observed in both wild type and HPV transgenic mice infected with *C. muridarum*. Cervical dysplasia scored as CIN II was detected in both infected groups (WT, 3.3 ± 0.3; K14-HPV-E7, 3.5 ± 0.3) but cervical tissues from the respective uninfected control groups were normal (WT, 1.3 ± 0.3; K14-HPV-E7, 1.8 ± 0.5) 127. While similar studies cannot be undertaken in humans, it is possible that future studies using human-derived cervical 128,129 or fallopian 125,126 epithelial cell or organ models in conjunction with low passage clinical isolates of known virulence or mutagenic potential will provide future insights.

## TREATMENT AND PREVENTION

Antibiotics effective against chlamydial infections cross host membranes and are active intracellularly. These target protein biosynthesis, primarily by interactions with the 50S or 30S ribosomal subunits. Antibiotics that target cell wall biosynthesis are also effective. The current recommendation of the CDC for treatment for uncomplicated genital infections in nonpregnant adolescents and adults is doxycycline for 7 days or azithromycin in a single dose 14. Azithromycin is the recommended first choice for treatment of pregnant women, with amoxicillin as alternative 14. Doxycycline and ofloxacin are contraindicated in pregnant women. Treatment for chlamydial infection in the context of PID is similar with the addition of a second-generation (cefoxitin) and all third-generation (ceftriaxone) cephalosporins for treatment of possible co-infection by other STI pathogens e.g. *Neisseria gonorrheae* 14. Treatment of LGV is more protracted (doxycycline 100 mg orally twice a day for 21 days) and may require aspiration/drainage to prevent ulcer formation. Sex partners should be evaluated, tested and treated. A test of cure is not recommended after completing treatment unless symptoms persist or if reinfection is suspected. However, testing sooner than 3-4 weeks post therapy completion may not be valid because of persisting, residual pathogen-derived nucleic acids 130,131. Treatment usually resolves infection but does not ameliorate preexisting inflammatory-mediated tissue damage. 

Although *in vivo* development of homotypic drug resistance has never been documented for *C. trachomatis*, spontaneous drug resistant mutants have been selected in cell culture or after passage with sub-inhibitory concentrations of drug (reviewed by 132). However, their reduced ability to infect in cell culture or in animal models of infection suggests that these mutations are associated with such significant metabolic compromise that they will be lost from a population in the absence of selection 133,134. Nevertheless, treatment failure has been described for individuals who completed a course of therapy and who were reportedly not at risk for reinfection 7,135. Mechanisms that could contribute to these clinical observations include the potential for ongoing infection in a drug-protected reservoir that facilitates autoinoculation after therapy 136 and/or changes in the metabolic or physiologic state of chlamydiae that alter sensitivity to antimicrobial treatment. Recent studies have revealed that long lasting *C. muridarum* colonization of the murine gastrointestinal (GI) tract can be established with very low inocula administered orally 137. Intravenous inoculation with a bioluminescent derivative of *C. muridarum* also resulted in GI colonization 138, revealing a systemic route to this mucosal site. Treatment with doxycycline cleared GI infection but azithromycin treatment was ineffective 139. Intriguingly, a very recent study revealed that GI-colonized female mice failed to auto-inoculate their genital tract 140. However, the extent to which colonization elicited or modulated a protective adaptive response was not reported. It is possible that protracted colonization may have induced an adaptive response that protected their reproductive tracts from infection. Analogies with rectal infection/carriage of *C. trachomatis* in women abound and have been extensively reviewed by Borel and colleagues 141. Protective immunity in humans is slow to develop and thus, women may be more vulnerable to reinfection after transmission to a treated partner via unprotected rectal intercourse or via auto-inoculation.

*C. trachomatis* development and replication *in vivo* may be subject to stresses imposed by nutritional requirements 142, innate and adaptive immune responses 143, host physiology via hormones 144,145,146 and even competition with commensals or co-pathogens 147,148. Conditions that delay bacterial multiplication impair effectiveness of antibiotics in many microorganisms 149,150,151. Asymptomatic cervical infection lasting up to four years has been documented 12 but it is not known if infection could have been detected throughout or if infection waxed and waned entering periods of persistence or dormancy. Conditions that arrest chlamydiae mid-cycle or promote aberrant forms influence antimicrobial sensitivity in cell culture 152,153. Prospective observational studies in women are unethical and the establishment of animal models of persistent infection has been challenging. Nevertheless, azithromycin failure is more frequent in the murine model in the context of amoxicillin-induced persistence 154. Failure was more frequent when azithromycin was administered as a single dose rather than distributed over a period of days, suggesting that it might be prevented by improved absorption or extended exposure to the drug. A study performed with 85 patients (men and women) with uncomplicated dual infection with *C. trachomatis* and *Mycoplasma genitalium* receiving an extended treatment regimen achieved an eradication rate of 98.8% 155, suggesting that this approach may be sufficient to limit therapy failures. Practical aspects related to patient compliance with prolonged therapy must also be balanced with the impact on potential co-pathogens such as *N. gonorrheae* and *M. genitalium*. STI treatment guidelines now advise the use of single dose azithromycin in combination with ceftriaxone for treatment of uncomplicated *N. gonorrheae* infection in an effort to preserve this antibiotic in the face of increasing resistance 14,156,157. Resistance to tetracyclines is increasingly prevalent in this STI, limiting the usefulness of doxycycline in this context. *M. genitalium*, an etiology for nongonococcal urethritis in men 158, is associated with cervicitis and PID in women 159,160. Doxycycline is ineffective against this pathogen and homotypic resistance to azithromycin is well recognized 161,162,163. Thus, anti-chlamydial therapy in co-infected patients could potentially select or fix resistant *M. genitalium* strains within the population 164. There is an increasing need to consider the development of new regimens or novel antimicrobials to treat polymicrobial STI.

Women with any of the following risk factors should be tested routinely for *Chlamydia*: mucopurulent cervicitis, sexually active and <20 years of age, >1 sex partner during the last 3 months, or inconsistent use of barrier contraception while in a nonmonogamous relationship 14. Public health measures have encouraged screening, treatment and barrier contraception for more than 20 years. Although minimal rates of screening coverage have yet to be achieved in vulnerable populations, reductions in PID incidence have been observed 165,166. However, simply expanding screening risks becoming cost ineffective 167 and early treatment may blunt the development of protective immunity 168.

Evidence for natural immunity in humans includes decreased prevalence with increasing age 169 and decreased infection concordance with increased age of sexual partnerships 7,170. IFN-γ-producing *Chlamydia*-responsive CD4 T cells are key mediators of protection 171,172 in mice and the relative ability of several candidate vaccine preparations to protect murine oviducts from disease correlated directly with their induction of CD4 T cell IFN-γ 173,174,175. Chlamydial proteins that induce CD4 and CD8 T cell production of IFN-γ in humans have been identified 176,177. Longitudinal analysis of PBMC responses to cHSP60 and EB conducted in sex workers revealed IFN-γ responses to cHSP60, but not to EB, were associated with protection from incident infection 178. Anti-chlamydial antibody contributes to resistance to reinfection 179. These may act indirectly by promoting T-helper 1 activation and cellular effector responses 180 because epidemiological studies associate high antibody titers with infertility 181 and do not correlate with infection resolution or control of ascending infection 109. 

Nevertheless, there is no evidence that natural immunity provides complete, long-term protection sufficient to prevent damaging immune pathology. Consequently, developing an effective vaccine is a highly desired, ambitious goal (reviewed by 182,183). Candidate vaccines against *C. trachomatis* have languished in preclinical testing but Phase I trials of chlamydial vaccine candidates are anticipated. Furthermore, advances in adjuvant development hold promise for additional candidates to enter clinical evaluation. MOMP is a highly abundant surface antigen that has long been considered a promising candidate. Novel formulations delivering this protein via cationic liposomes induced antibody, type-1 immunity and partial protection from infection in minipigs 184 and significant protection against upper tract disease in mice 185,186. Intranasal immunization using MOMP in combination with Nanostat^™^, oil-in-water nanoemulsion in mice after challenge 187. A polyvalent vaccine comprised of MOMP with PMPs formulated with DDA/MPL adjuvants reduces chlamydial shedding when tested in a transcervical *C. trachomatis* mouse model 188. Route of delivery has proven particularly important. Uterine vaccination with inactivated *C. trachomatis* complexed with charge switching synthetic adjuvant particles (cSAPs) linked with a TLR7-agonist, resiquimod, induced superior chlamydial clearance when compared to intranasal or intramuscular delivery because it elicited resident memory T cells in murine genital mucosa 189. This study highlighted the importance of investigating immunologic responses specific to the genital tract to determine optimal strategies for developing vaccines that elicit broad, long lasting protection against urogenital infection.
